# Aetiology of haemorrhagic cystitis: BK Polyomavirus and Adenovirus detection

**DOI:** 10.4102/sajid.v40i1.685

**Published:** 2025-02-11

**Authors:** Yoliswa Z. Chili, Menzi B. Nkosi, Nicky-Louise Byrne, Moepeng J. Maseko, Stephen N.J. Korsman, Gert U. van Zyl

**Affiliations:** 1 Department of Pathology, Division of Medical Virology, Faculty of Health Sciences, Stellenbosch University, Cape Town, South Africa; 2 Department of Pathology, Division of Medical Virology, Faculty of Health Sciences, National Health Laboratory Service, Tygerberg Hospital, Cape Town, South Africa; 3 Department of Pathology, Division of Medical Virology, Faculty of Health Sciences, University of Cape Town, Cape Town, South Africa; 4 Department of Pathology, Division of Medical Virology, Faculty of Health Sciences, National Health Laboratory Service, Groote Schuur Hospital, Cape Town, South Africa

**Keywords:** haemorrhagic cystitis, CLL, adenovirus, BK polyomavirus

## Abstract

This case report details haemorrhagic cystitis in a 49-year-old female with chronic lymphocytic leukaemia (CLL), recurrent haematuria and detected BK polyomavirus (BKPyV) and adenovirus (HAdV) in urine. During chemotherapy cycles, quantitative polymerase chain reaction (PCR) assays found high HAdV load, similar to a HAdV-B2 genotype 78, a rare genotype that is closely related to HAdV-11.

**Contribution:**

Viral interactions reveal immune system vulnerabilities.

## Case presentation

A 49-year-old human immunodeficiency virus (HIV)-negative female patient, known with chronic lymphocytic leukaemia (CLL), was referred for worsening haematuria. In 2020, she initially presented with disseminated lymphadenopathy, splenomegaly and a right adnexal mass. Subsequently, peripheral blood flow cytometry and CT scan imaging revealed a diagnosis of Rai stage 3 CLL of the B-cell. She has received three cycles of Fludarabine, Cyclophosphamide and Rituximab (FCR) since her diagnosis, with the last cycle being in March 2023.

Five weeks after receiving FCR chemotherapy, she began to experience worsening dysuria, frequency and urgency, macroscopic haematuria, suprapubic tenderness and diarrhoea. During this admission, she was initiated on intravenous antibiotics while conducting investigations to identify the causative agent. After diagnosis, intravenous fluids managed her condition, eliminating the need for bladder washes.

She previously presented with haemorrhagic cystitis in 2022, subsequent to the completion of FCR chemotherapy. She received hospital treatment, excluding antiviral therapy (ART), for this presentation and was subsequently discharged once her symptoms had resolved, without a definitive causative agent being identified at that time.

We used a qualitative HAdV polymerase chain reaction (PCR) during her current presentation to detect the presence of human adenovirus (HAdV) in her urine; urine microscopy, culture and susceptibility determined 3+ leukocytes, 3+ erythrocytes and no growth after 48 h. There were also no Schistosoma haematobium ova observed in the urine. No growth on stool culture determined either. In addition, we determined the urine BK polyomavirus (BKPyV) viral load to be 1749 copies/mL and the HAdV viral load to be 55 800 000 copies/mL. Sanger sequencing identified a rare HAdV-B2 genotype (P11H11F77). The hexon sequence obtained showed the highest similarity to HAdV-11, as identified by the Basic Local Alignment Search Tool (BLAST) search, but with features resembling the recombinant HAdV-78, which includes an HAdV-11-like penton base, an HAdV-11-like hexon, and an HAdV-77-like fibre. This sequence received GenBank accession number PQ539423. However, this cannot definitively confirm it as HAdV-78 without additional sequence data, such as the fibre region. These results are consistent with published reports identifying HAdV-11 as the HAdV type most often associated with haemorrhagic cystitis. Often detected in the urine of asymptomatic individuals, BKPyV can persist for an extended period of time. This, together with the much higher HAdV urine viral load than that of BKPyV, suggests that HAdV may have been the more important aetiology in the current acute presentation. However, BKPyV coinfection may have contributed to the pathogenesis of haemorrhagic cystitis.

## Research methods and design

We used the NucliSens easyMAG (BioMérieux, Marcy-I’Étoile, France) to extract a 100 µL aliquot of the original 500 µL urine patient sample. The next step was a PCR that targeted the hexon protein using a touch-down PCR with primers that were described by Khalaf et al.^1^ These primers were chosen as they target the hexon protein, located in the L3 region, which is one of the major coat proteins synthesised during late infection and is a major structural protein of the virus. We purified the PCR amplicons and cloned them using the pJET 1.2 blunt-end cloning vector from the CloneJET PCR cloning kit (ThermoFisher Scientific, Waltham, Massachusetts) through blunt-end ligation. Purified plasmids underwent Sanger sequencing using CloneJET sequencing primers (ThermoFisher Scientific, Waltham, Massachusetts). After the sequencing process, we trimmed the ends using Geneious Primer 2.0 (Dotmatics, Boston, Massachusetts, USA).

We evaluated sequence similarity using the BLAST from the National Centre for Biotechnology Information (NCBI). The sequences showed a 97.54% similarity to GenBank accession number KT970442.1, classified as a novel HAdV-B2 genotype-GenBank accession number PQ539423. This genotype, designated as HAdV-78, is recognised as an intertypic recombinant. This HAdV-78 was described to exhibit a HAdV-11-like penton base, a HAdV-11-like hexon, and a HAdV-77-like fibre (P11H11F77). This NCBI reference virus was isolated from two unrelated immunocompromised patients, both recipients of a haematopoietic stem cell transplants (HSCT). The first patient present with the HAdV-78 suffered from BKPyV and later HAdV, while the second patient primarily dealt with adenoviral complications. Both haematopoietic cell transplant recipients received cidofovir for viral infections; in contrast, our patient’s management focussed on supportive care and the administration of intravenous fluids, reflecting the various approaches to this clinical context. With respect to the overall clinical outcomes, the clinical trajectories for both haematopoietic cell transplants recipients were marked by multiple readmissions and progressive deterioration because of viral reactivations, leading to mortality within 150 days’ post-transplant.^2^

## Discussion

Haemorrhagic cystitis is defined by the presence of microscopic or macroscopic haematuria, which may or may not be accompanied by dysuria, micturition frequency and suprapubic pain, all in the absence of any confounding variables.^3^ Cystitis is bladder inflammation which may be caused by mechanical, radiative, infectious or chemical agents.^4^ The infective causes of haemorrhagic cystitis, which needed to be excluded in this patient, include bacterial, fungal and viral infections. The viral infections responsible for haemorrhagic cystitis include HAdV, BKPyV and cytomegalovirus (CMV) which affect immunosuppressed patients, including those with HIV or acquired immunodeficiency syndrome (AIDS), those on immunosuppressive agents and patients suffering from leukaemia because of their inability to produce functional leukocytes.^2^

BK polyomavirus is a double-stranded DNA virus that is ubiquitous in nature and has the potential to infect numerous species, including humans. It was first identified in the urine of a renal allograft recipient. It typically leads to tubulointerstitial nephritis, ureteric stenosis, nephropathy and haemorrhagic stenosis in immunocompromised individuals.^5^ High BKPyV viral loads (up to 10^7^ copies/mL in the urine), the presence of decoy cells with viral inclusions, and a plasma viral load of about 10^4^ copies/mL are suggestive of nephropathy. Major risk factors for nephropathy and subsequent haemorrhagic cystitis include an elevated BKPyV viral load, immunosuppression, HLA type, age, race and diabetes.^6^

Similarly, to BKPyV, HAdV is a double-stranded DNA virus that typically causes asymptomatic infection and possesses the ability to establish latency. However, there are multiple genotypes, some of which are associated with respiratory disease, keratoconjunctivitis, pharyngoconjunctival fever and haemorrhagic cystitis in the immunocompromised.^3^ Transmission of HAdV occurs through inhalation of respiratory secretions, fomites, faecal-oral transmission, direct conjunctival inoculation and exposure to contaminated blood and blood products.^3^ HAdV-induced haemorrhagic cystitis is associated with serotypes 7, 11, 34, or 35 and high viral load in the urine. However, no diagnostic viral load threshold has been established. Histology remains the diagnostic gold standard.^7^

Chemical cystitis, particularly when associated with cyclophosphamide-containing chemotherapy, can exacerbate haemorrhagic cystitis. Cyclophosphamide contributes to this condition through its liver metabolite, acrolein, which induces a pyroptotic reaction. This reaction leads to gradual ulceration of the urothelium, ultimately exposing the underlying vasculature. As the process unfolds, reactive oxygen species (ROS) are released, activating the nuclear factor Kappa B apoptotic pathway. This cascade results in the depletion of nicotinamide adenine dinucleotide (NAD) and adenosine triphosphate (ATP), causing a loss of cellular energy and eventual cell death. The inflammatory damage that ensues can progress to microscopic or macroscopic haematuria.^4^ This highlights the various risk factors present in our patient that contributed to her development of haemorrhagic cystitis.

Patients who are undergoing immunosuppressive treatment are susceptible to viral coinfection. Bil-Lula et al. discovered that BKPyV significantly upregulates HAdV expression via gene transactivation. Their study revealed that coinfected cells highly expressed BKPyV’s large and small tumour antigens, essential for replication, alongside HAdV’s E4 orf 1, enhancing HAdV replication. The mRNA levels of both viruses were elevated in coinfected cells, suggesting a synergistic effect.^5^

In this case, CLL was a contributing factor to the patient’s immunosuppression, which further contributed to BKPyV and HAdV reactivation. Chronic lymphocytic leukaemia is a lymphoproliferative disorder that is associated with hypogammaglobulinemia, B-cell dysfunction and T-cell abnormalities. When patients present with clinical manifestations of massive splenomegaly, lymphadenopathy, constitutional symptoms, anaemia and thrombocytopenia, we recommend treating CLL based on Rai staging. For minimal haematuria (Stage 0), supportive care with increased fluids and viral load monitoring is sufficient. In mild to moderate cases (Stages I–II), adjust immunosuppressive therapy and consider low-dose antiviral agents such as cidofovir. For severe haematuria or bladder compromise (Stages III–IV), escalate treatment with higher antiviral doses, immunosuppression reduction, and adjuvant therapies such as intravenous immunoglobulin (IVIG) or hyperbaric oxygen therapy. This approach aligns with international guidelines that recommend severity-based individualised treatment and careful immunosuppression management.^8^ Bacterial and viral infections afflict 30% to 50% of patients with CLL. The increased susceptibility to infection is a result of impaired B-cell function and T helper cell dysfunction in conjunction with the use of antineoplastic agents in patients suffering from CLL.^9^

The potential to mitigate the effects of concurrent CLL immunosuppression, chemotherapy immunosuppression and viral coinfection with BKPyV and HAdV – all of which simultaneously contributed to the development of haemorrhagic cystitis – is relevant. Sodium 2-mercaptoethane sulfonate (Mesna) may have been advantageous for the patient in order to neutralise the acrolein. Bladder irrigation is an additional approach to managing chemical cystitis, as it reduces urokinase levels, thereby reducing the risk of haemorrhage. In addition, hyperbaric oxygen utilisation is an expanding area of research because of its capacity to promote angiogenesis, induce vasoconstriction, enhance immune function and stimulate tissue granulation.^4^

Given its *in vitro* efficacy in treating both BKPyV and adenovirus, cidofovir would have been an ideal antiviral prescription for potential pharmacological therapies. Cidofovir, a monophosphate nucleotide analogue, competitively inhibits DNA polymerase-induced chain elongation.^10^ *In vitro* studies have shown potential prospects with regards to cidofovir, brincidofovir, ganciclovir and ribavirin for HAdV-induced haemorrhagic cystitis. Clinically, cidofovir is employed; nonetheless, its utilisation is constrained by difficulties in public sector accessibility. Consequently, bladder irrigation was conducted without the use of cidofovir. The patient in this case report has successfully resolved her haemorrhagic cystitis and has shown clinical improvement. Despite ribavirin demonstrating potential *in vitro*, its efficacy against severe HAdV infections is limited. Brincidofovir is a lipid-conjugated formulation of cidofovir that exhibits reduced nephrotoxicity and enhanced oral bioavailability, which has shown some promise *in vitro*.^6^ When it comes to treating BKPyV, the patient may have benefited from pre-emptive viral load monitoring with the withdrawal of immunosuppression. Numerous case studies have reported the benefit of cidofovir; however, no randomised control trials exist to corroborate this.^11^

## Conclusion

This case study highlights the development of haemorrhagic cystitis in a patient with CLL because of multiple factors, including compromised immune function and chemotherapy-induced immunosuppression. These factors reactivated latent BKPyV and HAdV, both recognised causes of haemorrhagic cystitis in immunocompromised individuals. Frequent diagnostic testing to identify infections and, where appropriate, viral load monitoring of persistent infections to detect incident infections or immune suppression-associated reactivation are crucial for patients undergoing immunosuppression, aiding timely adjustments in treatment to prevent severe complications. Furthermore, the global investigation of the molecular epidemiology of HAdVs implicated in haemorrhagic cystitis could reveal emergent genotypes and epidemiological trends.
